# The determinants of trichiasis recurrence differ at one and two years following lid surgery in Vietnam: A community-based intervention study

**DOI:** 10.4103/0974-620X.57311

**Published:** 2009

**Authors:** Rajiv Khandekar, Ton Tin K. Thanh, Vu Quoc Luong

**Affiliations:** British Columbia Center for Epidemiologic and International Ophthalmology, University of British Columbia, Canada; 1 National Institute of Ophthalmology, Ministry of Health, Hanoi, Vietnam

**Keywords:** *Lid surgery*, *recurrence*, *trachoma*, *trichiasisRecurrence*, *Vietnam*, Space bet trichiasis and recurrence

## Abstract

**Aim:**

To compare determinants for recurrence of trichiasis at one and two years following lid surgery in Vietnam.

**Study Design:**

Community-based intervention study.

**Methods:**

This study was carried out between 2000 and 2003 in four trachoma-endemic districts of Vietnam. Trained trichiasis surgeons performed modified Cuenod Nataf lid surgery on 648 eyes of 472 patients with Trachomatous trichiasis (TT). Trained investigators collected information on ocular and lid status before surgery and at one and two years following surgery. Trichiasis recurrence was calculated after adjusting for one or both eyes of each operated individual.

**Results:**

Fifty-six eyes developed recurrence at one year with adjusted prevalence of 8.8% (95% CI 6.60-11.01). One hundred and one eyes [15.9% (95% CI 13.04-18.72)] had recurrence two years following surgery. Female gender, older age group, study area, severe grade of trachomatous scarring (TS), past history of lid surgery, postoperative suture adjustment and surgeon were risk factors for recurrence at the end of one year. Study area and previous lid surgery were risk factors for recurrence in the second year. Recurrence at one year could be predicted if study area and severity of Trachomatous Scarring (TS) are known.

**Conclusions:**

One and two year recurrence rates with modified Cuenod Nataf lid surgeries for TT in Vietnam were acceptably low. Early recurrence could be reduced by proper case selection. However, late recurrence seems to be dependent on interaction of risk factors. Only age of the patient was the reliable predictor of recurrence.

## Introduction

Low uptake of Trachomatous trichiasis (TT) surgery has always been of concern for the success of the ′S′ (surgery) component of the SAFE trachoma-control strategy.[1] Recurrence of trichiasis following intervention is a major barrier for the better uptake of TT surgery.[2]

The World Health Organization (WHO) has recommended Bilateral Tarsal Rotation (BTR) lid surgery to manage TT. Short and long-term recurrence rates of BTR range from 20 to 56% in trachoma-endemic countries.[3‐5] Many studies have been undertaken to estimate the magnitude of TT and risk factors of recurrence following different surgical procedures.[5‐8] Another procedure, known as Cuenod Nataf surgery is easy, feasible at the primary level, affordable to low-income population and is therefore practiced in many countries.[9] Trichiasis surgeons in Vietnam have modified this technique. In a study evaluating recurrence of TT after modified Cuenod Nataf procedure in Vietnam, authors reported a one year recurrence of 10.8%; two years following this lid surgery, recurrence rate was found to be 19%.[7] Although these rates were low, to further reduce it, determinants of short and long-term outcomes of this surgical procedure would be useful.

Different factors related to providers and receivers of TT management have been considered as risk factors [9‐11] However, very limited information on the risk factors of TT recurrence in relation to time elapsed after lid surgery is available in the literature. We present recurrence rates and risk factors at second year and compared them with the first year after surgery. This report is part of the Trachomatous Trichiasis Operational Research (TTOR) study in Vietnam from 2001 to 2003 and was supported by the International Trachoma Initiative (ITI).

## Materials and Methods

This was a community-based clinical intervention study. Ethical clearance was obtained from the Ministry of Health of Vietnam for this project. Written consents of participants were obtained.

Four hundred and seventy two patients with TT were recruited from four districts of three trachoma-endemic provinces of Vietnam. Trained field investigators evaluated the ocular status, visual status and trichiasis in each eye. The evaluation was carried out prior to surgery and one and two years after surgery. Standard tools for assessment such as distant vision acuity chart, ophthalmic loupe and torchlight were used. Past history of lid surgery was also inquired. Prior to surgery, the tarsal conjunctiva was examined under magnification. ′Trachomatous Scarring′ (TS) was graded mild if the conjunctiva showed the scars and blood vessels in the central tarsal area. If scarring obscured the conjunctival vasculature, the TS were considered to be of moderate grade. If the lid margin showed entropion and the tarsal plate was distorted, TS was labeled as ′severe′. Variables related to preoperative status of eyes, assessment methods and operative details are given in earlier publications.[6,12]

**Figure 1:**
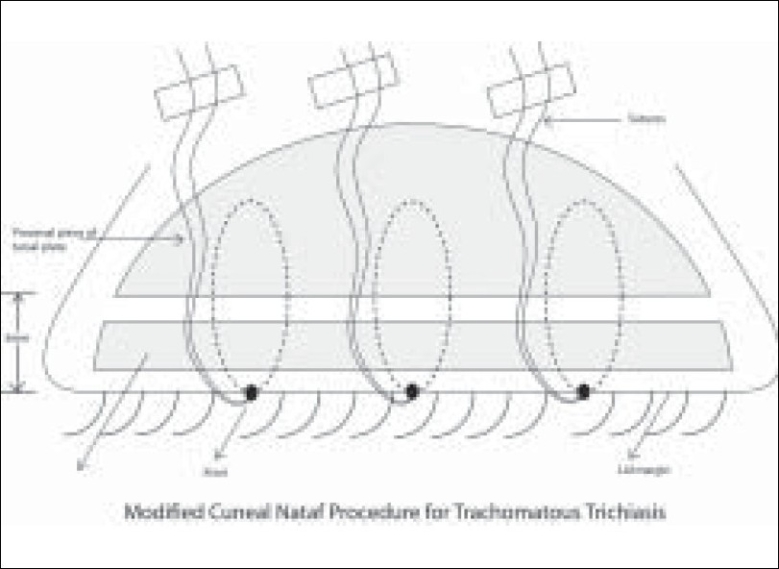
Modified Cuenod Nataf lid surgery procedure for trachomatous trichiasis in Vietnam (Schematic)

Eight experienced trichiasis surgeons (ophthalmologists) performed the modified Cuenod Nataf surgery to correct trichiasis in 648 eyes. The steps of lid surgery adopted in our study are simple [Figure 1]. An incision is made at the grey line of the lid margin of the upper lid. Muscle and fibrous tissue are separated from the front surface of the tarsus plate by blunt dissection in the upper and lateral edges of the plate. The tarsal plate is then fractured horizontally leaving a 3 mm wide distal portion. In the conventional procedure, a triangular piece of tarsal plate is removed at both ends of the plate. In the modification adopted in Vietnam, this procedure is not carried out. Three equidistant mattress sutures are placed along the width of the lid. These sutures are then passed from the lid margin, through the cut edge of the distal piece into the anterior surface of the proximal piece of the tarsal plate near the cut edge, then back through the anterior surface of the proximal piece emerging at the cut edge and finally through the cut edge of the distal piece, emerging at the lid margin. During the entire procedure, hemostasis is maintained. Ends of the sutures are tied with one overhand throw, left long and taped to the brow. Antibiotic ointment is applied to the upper lid, the wound and then eye is lightly patched. After 24 h, the correction is noted. If TT correction is excessive, knots are loosened and if correction is less than desired, the knots are pulled tighter. Sutures are removed on the seventh postoperative day. In the absence of infection or severe inflammatory response, no systemic antibiotic or antiinflammatory medications are given.

Presence of one eyelash in the operated eye on follow-up examinations was considered as recurrence of TT. The examination was carried out one week, six weeks, six months, one year and two years following surgery. If recurrence was noted within one week of surgery, it was termed as failure of surgical procedure. Since two eyes with surgical failure were corrected within six weeks of intervention, we included them in the longitudinal study and the presence of trichiasis in each eye was considered as a recurrence.

We piloted the study method and data collection form in one village, which was not included in the study. We audited the completed forms in the villages and then computed the data using EPI6 software (CDC, Atlanta). A unique identification code was given to each person and files of baseline, one year and two years follow-up were merged. A person was listed twice if both eyes were operated for TT.

Statistical analysis of study date was performed using Statistical Package for Social Studies (SPSS – 9) by the parametric method of univariate type of analysis. The recurrence rates per eye at the end of one and two years was calculated. To minimize the effect of bilateral surgeries, weighted outcomes were used if both eyes of the same person were operated. SPSS software was used for this purpose. The frequencies of recurrence, percentage proportions and 95% confidence intervals were calculated. Binary logistic regression analysis was carried out. Adjusted Odd′s ratios for recurrence were calculated using step-in method. Trachomatous trichiasis recurrence was the dependent variable while age, gender, past history of lid surgery, adjusting sutures within one week of surgery and grade of the healed trachoma were the independent variables. Unilateral and bilateral surgeries of the same person were also considered in regression analysis.

Participants found to have active trachoma at the time of surgery or follow-up examination were treated with azithromycin. Misdirected lashes were epilated for immediate relief, and patients were subsequently offered repeat surgery at no cost.

In our cohort, 926 eyes of 472 patients were included. Of them, 648 eyes with TT were operated. We examined 761 eyes of 463 patients at one year follow-up and 636 eyes of 453 patients two years after surgery. The remaining patients had either died or left the study area. For the present study, information of 636 eyes at baseline, one year and two years after surgery were used to review risk factors of recurrence.

Frequencies and recurrence rates per eye are given in Table 1. Recurrence rate per eye at one year following modified Cuenod Nataf surgery after adjusting for both eyes undergoing TT surgery was 8.87% (95% CI 8.81–8.93). Severity of TS, past history of lid surgery and suture adjustment within first week following surgery were significantly associated with the recurrence at one year.

**Table 1:** Recurrence of Trachomatous Trichiasis following modifi ed Cuenod Nataf lid surgery in Vietnam

*Variant*	*Baseline*	*1 year after surgery*			*Between 1 and 2 years*			*Cumulative recurrence at 2 years*		
		#	%	*Statistical Validation*	#	%	*Statistical Validation*	#	%	*Statistical Validation*
Gender										
Male	157	9	5.7	RR = 1.17	10	6.8	RR = 1.2	19	12.1	RR = 1.41
Female	479	47	9.8	(0.86–3.41)	35	8.1	(0.61–2.36)	82	17.1	(0.89–2.25)
Age-group
<50	31	0	0.0	X^2^ = 11.8	1	3.2	X^2^ = 4.55	1	3.1	X^2^ = 5.58
50 to 59	89	3	3.4	Df = 2	8	9.3	Df = 3	11	12.4	Df = 3
60 to 69	138	7	5.1	P = 0.03	15	11.5	P = 0.2	22	15.9	P = 0.2
70 & +	376	46	12.2		21	6.4		67	17.8	
Missing	2
Study area
Vinh Phuc	157	10	6.4	X^2^ = 6.52	12	8.2	RR =	22	15.9	X^2^ = 35.2
Ha Nam	176	19	10.8	Df = 3	0	0.0	2.25	19	9.1	Df =
Thanh Hoa (Tho	100	4	4.0	P = 0.09	0	0.0	(1.2–4.2)	4	4	P =0.1 × 1-4
Xuan)	230	23	11.3		33	18.3	P = 0.012	56	27.6	
Thanh Hoa (Vinh Loc)
Site of TT					30			71		
Medial 1/3	393	41	10.4	X^2^ = 5.71	12	8.5	X^2^ = 2.97	16	18.1	X^2^ = 2.26
Middle 1/3	119	4	3.4	Df = 2	2	10.4	Df = 2	9	13.4	Df = 2
Lateral 1/3	71	7	9.9	P = 0.06	1	3.1	P = 0.2	20	12.7	P = 0.32
Missing	53	4
Trachomatous
Scarring	183	9	4.9	X^2^ = 14.9	11	6.3	X^2^ = 3.83	21	11.5	X^2^ = 15.9
Mild	249	16	6.4	Df = 2	15	6.4	Df = 3	21	12.4	Df = 2
Moderate	200	30	15.0	P <0.005	19	11.2	P = 0.15	49	24.5	P = 0.0002
Severe	4							10		
Missing
Lid surgery in the Past							RR = 2.08			
No	472	32	6.8	RR = 2.2	28	6.8	(1.2-3.2)	60	12.6	RR = 2.02 (1.4–2.87)
Yes	161	24	14.9	(1.34–2.64)	17	14.2	P = 0.02	41	25.5	P = 0.001
Missing	3			P = 0.001						
Suture adjustment				RR = 1.45			
No	500	36	7.2	RR = 2.2	32	6.9	(0.72-2.9)	26	24.3	RR = 1.75(1.2-2.67)
Yes	107	17	15.9	(1.3-3.8)	9	10.0	p = 0.4	68	13.6	P=0.008
Surgeon				p = 0.0006
1	89	8	9.0		17	21.0		25	30.3	
2	100	4	4.0		0	0.0		4	4	
3	58	6	10.3	X^2^ = 9.98	12	23.1		18	31	X^2^ = 42.65
4	56	9	16.1	Df = 7	2	4.3	-	11	19.6	Df = 7
5	96	6	6.2	P < 0.2	7	7.8		13	13.5	P = 0.7 × 10 −4
6	61	4	6.6		8	14.0		12	19.7	
7	103	13	12.6		0	0.0		6	8.2	
8	73	6	8.2		0	0.0		6	8.2	
Total	636	56	8.8	6.60-11.01	45	7.8	4.98-9.64	101	15.9	13.04-18.72

To calculate recurrence after one year, we deducted the number of eyes with recurrence in the first year. Thus the denominator for recurrence after the first year was 580 eyes. Between the first and second follow-up, another 45 eyes developed recurrence with an adjusted recurrence rate of 7.96% (95% CI 7.91–8.02). In this group, the study area (Vin Loc district of Thanh Hoa province), past history of lid surgery and age were risk factors for recurrence. The cumulative recurrence rate per eye at two years following modified Cuenod Nataf surgery was 15.9% (95% CI 13.04–- 18.72). Operating surgeon, severity of trichiasis, past history of lid surgery and postoperative suture adjustments were significantly correlated to recurrence of TT at two years.

Recurrence of TT in relation to time following modified Cuenod Nataf lid surgery is given in Table 2. Differences of recurrence for variables were calculated using the difference of recurrence in year one and year two. We also calculated differences in recurrence among subgroups (e.g. male versus female). The mean age of persons with recurrence at one year was 67.8 years (SD 10.5 years), while the mean age of persons without recurrence at two years was 64.1 years (SD 11.2 years). The age of persons in recurrence group was 3.8 years more than non-recurrence group (95% CI 3.62–3.87). Trachomatous trichiasis recurrence was higher in year one compared to year two. In the Vin Phuc area, a marginal increase in recurrence rate in the second year was found. In all other study areas, the rate in year one was higher than year two. Those eyes with a mild grade of healed trachoma scar at time of surgery had higher recurrence rates in year two compared to year one following surgery. Eyes having TT in the middle one-third of the lid had higher recurrence in the year two compared to year one after surgery. The difference of TT recurrence in one and two years among eyes suggested that recurrence was more common within first year than in the second year. Eyes that had lid surgery in the past had higher risk of recurrence compared to the eyes not operated in the past irrespective of time lapsed following the lid surgery procedure. Similarly, recurrence in eyes in which sutures were adjusted within one week of surgery had higher risk of recurrence in year one than in the year two. Trachomatous trichiasis recurrence two years after surgery also varied with the operating surgeon.

**Table 2:** Comparison of TT recurrence by time among variables of TT

	Weighted* recurrence % in year one			Weighted* recurrence % in year two			*Difference of recurrence % in two Groups*	*Difference within subgroups of variables*
	%	95% CI		%	95% CI			
Gender								
Male	7.2	6.99	7.42	6.0	5.80	6.17	1.22	
Female	11.0	10.87	11.05	9.4	9.33	9.48	1.56	0.3
Age-group								
<50	0.7	0.62	0.87	11.2	10.74	11.65	-10.45	
50 to 59	3.8	3.62	4.03	14.2	13.83	14.58	-10.38	0.1
60 to 69	8.5	8.34	8.65	12.1	11.89	12.26	-3.58	6.9
70 & +	16.2	15.97	16.42	18.6	18.35	18.82	-2.39	8.1
Study area								
Vinh Phuc	5.5	5.33	5.66	11.3	11.10	11.56	-5.83	
Ha Nam	9.0	8.83	9.22	0.0			9.03	14.9
Thanh Hoa (Tho Xuan)	3.6	3.25	3.90	0.0			3.57	9.4
Thanh Hoa (Vinh Loc)	14.1	13.89	14.32	17.8	17.54	18.02	-3.67	2.2
Trachomatous Scarring								
Mild	2.8	2.60	2.96	9.9	9.64	10.11	-7.10	
Moderate	4.9	4.80	4.96	6.6	6.45	6.70	-1.70	5.4
Severe	22.5	22.21	22.83	9.4	9.27	9.61	13.08	20.2
Past history of lid								
surgery								
No	6.6	6.58	6.72	7.3	7.19	7.33	-0.61	
Yes	15.7	15.41	15.94	12.3	12.10	12.57	3.34	3.9
Site of TT before surgery								
Medial 1/3	10.9	10.77	10.97	9.7	9.63	9.82	1.15	8.0
Middle 1/3	3.7	3.49	3.88	10.6	10.23	10.88	-6.87	
Lateral 1/3	10.5	10.00	11.05	4.0	3.66	4.34	6.53	13.4
Surgeon								
1	7.6	7.24	8.03	25.6	24.97	26.27	-17.99	
2	3.6	3.30	3.97	0.0			3.64	21.6
3	11.7	11.03	12.37	26.5	25.59	27.43	-14.80	3.2
4	20.0	19.13	20.87	1.4	1.13	1.64	18.61	36.6
5	6.4	6.10	6.72	7.5	7.20	7.87	-1.12	16.9
6	7.8	7.37	8.28	13.2	12.63	13.78	-5.38	12.6
7	10.1	9.73	10.39	0.0			10.06	28.0
8	6.8	6.41	7.26	0.0			6.84	24.8
Suture adjustment								
No	7.0	6.89	7.02	8.1	7.99	8.13	-1.11	
Yes	16.8	16.46	17.23	9.4	9.13	9.73	7.41	8.5
Total	8.87	8.81	8.93	8.74	8.68	8.79	0.13	

* We weighted the recurrence based on the one eye and two eyes of the same person was operated to manage trachomatous trichiasis.

** While calculating the percentage of recurrence in 1 to 2 years we subtracted eyes that had recurrence in 1^st^ year from the denominator.

Prior to surgery 505 eyes had vision better than 6/60 and 111 eyes had vision less than 6/60. At one year following surgery, 25 (22.5%) blind eyes had recurrence and 31 (6.1%) ′Not blind′ eyes had recurrence. Recurrence of TT was significantly associated with blindness one year following surgery [RR = 3.67 (95% CI 2.26–5.06)]. Two years following surgery same 25 (22.4%) eyes were blind. Seventy-three eyes (14.5%) without recurrence were blind. [RR = 1.42 (95% CI 0.98–2.05)].

The regression analysis was carried out for the recurrence of trichiasis in the first and second year by using recurrence as dependent variable and with step-in method. We tested variables like age, gender, past history of lid surgery, suture pulled postoperatively and severity of scarring in the lid [Table 3].

**Table 3:** Predictors of recurrence in eyes at 1 year 8 2 years following modifi ed Cuneod Nataf surgery

*Predictor*	*Variants*	*Recurrence at 1 year*				*Recurrence at 2 year*			
		Adjusted Odd's ratio*	*95% Confidence Interval*		*P value*	Adjusted Odd's ratio*	*95% Confidence Interval*		*P value*
Gender	Male	1.003	1.99		0.99	0.33	0.11	0.99	0.05
	Female	1	1			1				

Age		0.98	0.96	1.01	0.21	1.09	1.04	1.14	0.0002

Past history of lid	No	0.72	0.38	1.38	0.33	0.71	0.35	1.47	0.36
surgery	Yes	1				1			

Trachomatous Scarring	Mild	0.63	0.31	1.27	0.63	0.63	0.31	1.27	0.63
	Moderate	0.39	0.19	0.81	0.39	0.39	0.19	0.81	0.39
	Severe	1.00				1			

Sutures adjusted after surgery	Yes	0.78	0.38	1.58	0.49	0.28	0.13	0.60	0.001
	No	1

Study site	Vinh Phuc	0.50	0.25	0.99	0.05	0.53	0.21	1.34	0.18
	Ha Nam	0.00			0.99	0.62	0.29	1.34	0.22
	Thanh Hoa (Tho Xuan)	0.15			0.005	0.19	0.02	1.64	0.13
	Thanh Hoa (Vinh Loc)	1	0.04	0.56		1			

Surgery in one eye
Surgery in both eyes		0.74	0.39	1.40	0.35	0.89	0.42	1.91	0.77
eyes		1

* Adjusted odd's ratio is calculated after weighing for one and both eyes of same person operated and using denominator as eyes not having recurrence in the 1st year. We used binominal regression analysis.

## Discussion

This study is perhaps the first one to identify the predictors of TT recurrence following modified Cuenod Nataf lid surgery. Outcome of our study may be compared to the recurrence rates reported after other surgical procedures. Trichiasis surgeons should understand risk factors of recurrence and focus on them at different times to improve success rates.

A longitudinal study, which provides information on operated eyes at baseline, one year and two years following surgery, makes our study more robust compared to the cross-sectional data of BTR procedure.[10]

One and two year recurrence rates with modified Cuenod Nataf lid surgeries performed to manage TT in endemic areas of Vietnam were acceptably low. The surgery involving simple steps, which can be practiced by trichiasis surgeons in a primary health setting, is a distinct advantage for its wider use in trachoma-endemic countries with limited availability of skilled surgeons. The literature has detailed short-term recurrence after one year and long-term recurrence after more than two years following BTR.[3,5]In our study, we used the modified Cuenod Netaf procedure. To our knowledge, factors responsible for short- and long-term recurrence following this procedure have not been studied. Further studies and longer follow-up are required to note if recurrence rates increases with time.

Interestingly, the original Cuenod Nataf procedure, which is widely practiced in Myanmar, has an additional step of cutting ′V′-shaped edges of the tarsal plate at both lateral ends. In Vietnam, a modification of the original surgery, which excludes cutting the lateral tarsal margins is employed. In our study, recurrence at the edges was still minimal.

The acceptability of surgery among providers mainly depends on ease of surgery, low cost and ability of trained person to perform the surgery in make-shift operation theatres in remote areas. However, patients look for a symptom-free situation following intervention. Knowing the predictors of surgical recurrence can help in selecting cases with possibility of better outcomes and improve acceptance. Such patients with higher risk of recurrence could be allotted to senior and experienced surgeons and guarded prognosis may be offered.

Difference in recurrence rates in first and second year after surgery could be due to continuation of scarring process in tissues of the upper lids with age. Limited amount of research in the past focused on slowing the progress or identifying the accelerating factors.[13] Hence, further research to review factors affecting scarring of lid tissue, before and after surgical intervention, would be of interest to trachoma scientists.

Surgical skills of operating surgeons are responsible for their success. Over- and under-correction of tarsal segment apposition in our study could be indirect indicators of surgical failure. However, tissue responses to surgery could differ in patients and that could also result in over and under correction. In two eyes within week one following surgery, we noted trichiasis in the lateral one-third of the lid and had loose sutures. Surgeons repaired them.

Scarring processes in Vietnamese people seems to differ from people of other trachoma-endemic areas. Locations of TT[14] and low prevalence of active trachoma in the Vietnamese community in recent years [15]seem to differ from that noted in the African[8,16,17] and Arab communities.[3,18] It should be noted that surgical procedures adopted to the above-mentioned places are mainly the WHO-recommended BTR procedures. Surgeons in Vietnam had attempted BTR procedures in the 1970s, but switched to modified Cuenod Nataf procedure due to its low recurrence rates and because non-ophthalmic health staff could also learn it fast and perform high quality surgeries in remote areas of the country. Therefore, recurrence and their predictors should be limited to the Vietnamese population and specific procedure of lid surgery that is adopted in last two decades and should be compared with other procedures carried out in other trachoma-endemic areas with caution.[7] Differences in outcomes of different studies could be due to genetic/ethnic differences and complexity of the variables involved.

Although our longitudinal study provided predictors of recurrence, a clinical intervention trial to compare outcomes of modified Cuenod Nataf procedure to other lid surgeries to treat TT would provide more dependable evidence.

Use of Azithromycin before and soon after TT surgery has been documented to reduce the recurrence of trichiasis.[19,20] For ethical reasons, cases identified with active trachoma were given single dose of Azithromycin, but distribution was not documented. Hence, we could not study its impact in reduction of recurrence of TT.

To summarize, risk factors for short- and long-term recurrence of trichiasis following of the modified Cuenod Nataf surgery practiced in Vietnam was studied. Among patients with a severe grade of TS, specific study areas, variation of operating surgeon, past history of lid surgery and suture adjustments in the immediate postoperative period were associated with a high risk of TT recurrence. However, interaction of different risk factors did not permit us to identify the predictors of recurrence of TT in first year and in the second year of surgery. Further studies with larger sample could explain the interaction of risk factors causing the recurrence.
